# Maintaining Genome Stability in Defiance of Mitotic DNA Damage

**DOI:** 10.3389/fgene.2016.00128

**Published:** 2016-07-21

**Authors:** Stefano Ferrari, Christian Gentili

**Affiliations:** Institute of Molecular Cancer Research, University of ZurichZurich, Switzerland

**Keywords:** cancer therapy, checkpoint, DNA damage, mitosis, phosphorylation, ubiquitylation

## Abstract

The implementation of decisions affecting cell viability and proliferation is based on prompt detection of the issue to be addressed, formulation and transmission of a correct set of instructions and fidelity in the execution of orders. While the first and the last are purely mechanical processes relying on the faithful functioning of single proteins or macromolecular complexes (sensors and effectors), information is the real cue, with signal amplitude, duration, and frequency ultimately determining the type of response. The cellular response to DNA damage is no exception to the rule. In this review article we focus on DNA damage responses in G2 and Mitosis. First, we set the stage describing mitosis and the machineries in charge of assembling the apparatus responsible for chromosome alignment and segregation as well as the inputs that control its function (checkpoints). Next, we examine the type of issues that a cell approaching mitosis might face, presenting the impact of post-translational modifications (PTMs) on the correct and timely functioning of pathways correcting errors or damage before chromosome segregation. We conclude this essay with a perspective on the current status of mitotic signaling pathway inhibitors and their potential use in cancer therapy.

## Introduction

Signaling pathways have been initially depicted as linear cascades, with elements organized in a hierarchical manner and unidirectional arrows connecting a stimulus to the final response through a defined number of intermediates (Rodbell, 1980). The advent of systems biology, following completion of animal and plant genome sequencing, has changed this view. The amount of information available today allows to more realistically depict signaling pathways as networks, where the arrangement of components (nodes) is such that some are more connected than others in a so-called scale-free topology, and where sets of components are organized in modular fashion, with a clear hierarchy among modules (Barabási and Oltvai, 2004). Such architecture has been shown to ensure fault tolerance (robustness) in response to challenges (Barabási and Oltvai, 2004; Zhu et al., 2007). Corollary to system-level approaches has been the development of mathematical models where the fluctuation of variables as it actually occurs in defined biological systems can be computed, hence realistically representing the dynamic flow of information in signaling networks (Samaga and Klamt, 2013; Gerard et al., 2015).

The descriptive power of systems biology and its ability to predict scenarios do not, however, dwarf the contribution of reductionism when it comes to identification of network components and to dissection of their molecular mechanism of action, including elucidation of the inputs that affect their sub-cellular localization, the interaction with partner proteins and biochemical properties such as stability and enzymatic activity. It is only thanks to the wealth of information provided by reductionist approaches that rational design of small molecule inhibitors able to interfere with the correct functioning of networks could be successfully guided (Asghar et al., 2015). Since the constitutive elements of network modules hierarchically relate to each other, modification of structural or enzymatic traits of one or more elements in a network will necessary affect network properties and result in outputs that are directly observable. Protein post-translational modification (PTM), in form of covalent addition of chemical groups or entire peptidyl moieties to one or more amino acids of a protein target, is the means to rapidly and, in most cases, reversibly affect such traits. The hierarchical, synergistic or antagonistic combination of PTMs defines a code that translates into distinct outputs, hence contributing to shape the emergent properties of complex systems like living organisms (Lorenz et al., 2011).

In this review, we focus on mitosis and examine how DNA damage occurring during transition through mitosis is addressed to avoid genome instability. Special emphasis will be set on the impact of PTMs on mechanisms of genome surveillance. We conclude with an up-to-date perspective on drugs designed for therapeutic purposes and that entered clinical trials.

## Mitosis and checkpoints

Transition through the cell cycle sets the conditions for cell division. This results in the generation of two daughter cells genetically identical to the mother, according to a principle originally formulated by Rudolf Virchow who first made such observation in 1858 and stated that every cell derives from a pre-exiting cell, “*omnis cellula e cellula”* (Mazzarello, 1999). The major events characterizing transition through the cell cycle are cell growth, by which means cells increase their size and the number of organelles, and duplication of genetic material in S-phase. If not perturbed, upon completion of DNA replication cells enter mitosis, a term that originally described nuclear division (Mazzarello, 1999). Perturbations of this program may be caused by external agents such as ionizing radiation or certain chemotherapeutic drugs as well as by endogenous metabolic processes, leading to the formation of double-strand breaks (DSBs). Inappropriate repair of DSBs may cause gross chromosomal aberrations, the activation of oncogenes or the inactivation of tumor suppressor genes resulting in carcinogenesis. Direct demonstration of the importance of surveillance pathways in the maintenance of genome stability (Hanahan and Weinberg, 2011) is provided by genetic conditions characterized by dysfunction of the machinery that signals DNA damage and/or addresses its repair, which are associated with a predisposition to the development of cancer (Curtin, 2012).

### Mitosis

Mitosis is probably the most spectacular event a cell undergoes to during its lifetime and it is essentially the process by which the duplicated genetic information is equally distributed to the daughter cells. Morphological changes that are easily observable with a microscope allow distinguishing sub-phases of mitosis consisting of prophase, metaphase, anaphase and telophase. These are followed by cytokinesis, ultimately causing physical separation of the daughter cells. The use of suitable model organisms and the support provided by modern technology has led us to a deep understanding of mechanistic aspects and regulatory pathways controlling the onset, execution and completion of mitosis. Briefly, in S-phase newly synthesized DNA emerging behind replication complexes that processively move on template DNA is maintained catenated throughout its length by ring-shaped cohesins and sister chromatids are held together at the centromeric region where kinetochores have been assembled (Kenney and Heald, 2006; Walczak et al., 2010). As cells move to prophase, chromatin condensation takes place, leading to the formation of visible rod-shaped structures, with a reduction of the length of DNA to an extent compatible with the distance that chromatids cover when moving to the opposite poles of the mitotic spindle (Walczak et al., 2010). Chromatin condensation results from the action of a multi-subunit protein complex called condensin, whose recruitment and activity are positively controlled by phosphorylation through CDK1, Aurora-B and PLKs and opposed by phosphorylation through CK2 (Hirano, 2012). Topoisomerase II, which undergoes phosphorylation and sumoylation in mitosis (Dephoure et al., 2008; Hendriks et al., 2014), ensures decatenation of sister chromatids prior to condensation (Hirano, 2015). Segregation of compacted chromosomes is initially prevented by cohesins (Peters et al., 2008) that are controlled by a combination of PTMs at lysine residues involving acetylation and sumoylation (Rudra and Skibbens, 2013) and are first removed at chromosome arms during prophase through PLK1-mediated phosphorylation (Hauf et al., 2005). At this time centromeric regions are protected by the protein shugoshin that, through recruitment of the phosphatase PP2A, counteracts PLK1 activity (Kitajima et al., 2006; Liu et al., 2013b). Construction of the mitotic spindle is the necessary step for physical separation of chromatids, with different strategies employed in distinct organisms to promote microtubule-to-kinetochore contacts (Boettcher and Barral, 2013). Microtubules forming the cell's cytoskeleton are disassembled in late prophase and highly dynamic microtubules radiate at this point from mature centrosomes or self-organize around chromosomes (Heald et al., 1996, 1997; Karsenti and Vernos, 2001), driving migration of centrosomes to opposite poles of the cell (inter-polar microtubules), anchoring centrosomes to the plasma membrane and positioning the spindle (astral microtubules) and initiating the capture of chromosomes (kinetochore microtubules). All these events are controlled by mitotic kinases (Nigg, 2001; Walczak et al., 2010).

In prophase, more than 100 proteins assemble around each centromeric region forming the kinetochore, while in the cytoplasm pairs of centrioles that have duplicated during S phase remain linked together at the proximal ends by a proteinaceous link containing C-Nap1 and rootletin, which is removed at mitotic entry through NEK2-mediated phosphorylation (Bahe et al., 2005; Hardy et al., 2014). Microtubule-chromosome interactions are characterized by the dynamic process of capture and release of erroneous attachments, as for instance merotelic attachments, which defines the condition of a single kinetochore being attached to microtubules nucleated from opposite spindle poles. Such interactions are principally regulated by Aurora-B-mediated phosphorylation of kinetochore components (Cheeseman, 2014), occur in prometaphase and metaphase, and largely affect the duration of these sub-phases (Pereira and Maiato, 2012). The subsequent chromosome congression to the spindle equator (metaphase plate) is coordinated by the action of motor proteins such as dynein and CENP-E, the latter being controlled by an Aurora-A/PP1-dependent phosphorylation switch (Kim et al., 2010), and is followed by a process called bi-orientation, where kinetochores of sister chromatids attach to microtubule bundles that have nucleated from opposite centrosomes (Tanaka et al., 2005). Upon congression of all sister pairs to the metaphase plate, licensing of a multimeric E3-ligase, the anaphase promoting complex/cyclosome (APC/C), ensues and leads to ubiquitylation and degradation of proteins such as Cyclin B, switching off CDK1 activity, and securin, freeing the enzyme separase that is now able to cleave and remove centromeric cohesins (Sivakumar and Gorbsky, 2015). This point marks the metaphase-to-anaphase transition where mechanical processes, consisting of inter-polar microtubule elongation and kinetochore microtubule shortening, as well as biochemical events mediated by the action of APC/C, determine the movement of chromatids to spindle poles (Castro et al., 2005; Goshima and Scholey, 2010). The process is completed by re-establishment of the nuclear membrane around decondensing chromosomes at telophase and is followed by physical separation of daughter cells, or cytokinesis (Pines and Rieder, 2001), assisted by the action of an acto-myosin contractile ring (D'Avino, 2009).

### G2/M checkpoint

**Figure 1:**
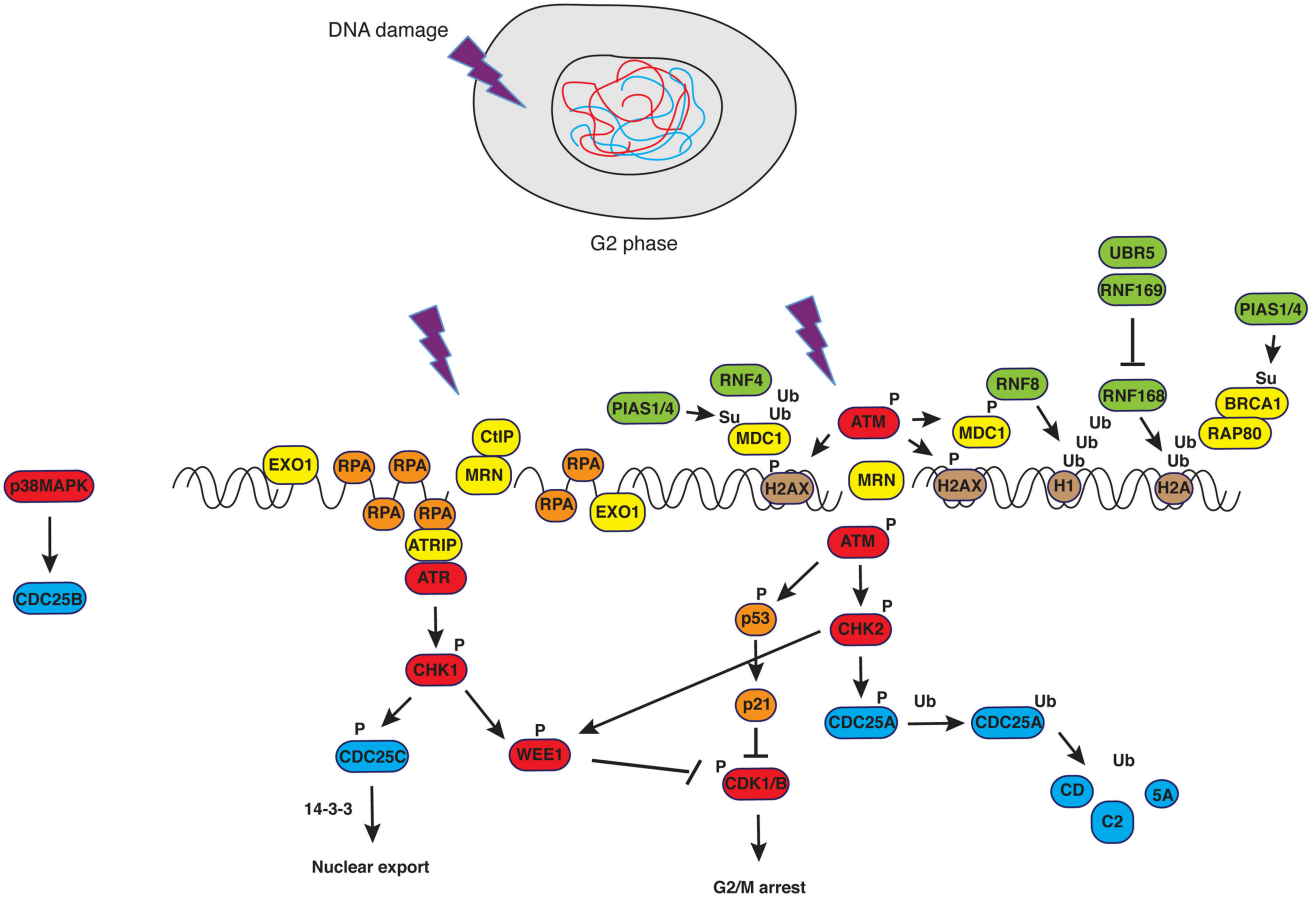
**DNA damage response in G2**. Upon generation of double strand breaks (DSBs), ATM is recruited to DNA ends in a MRN-dependent manner. Phosphorylation of H2AX creates epitopes facilitating the recruitment of DNA damage signaling and repair factors in a manner that depends on PTMs such as ubiquitylation and sumoylation (see text for details and Bologna and Ferrari, 2013). Successful activation of ATM-dependent signals causes controlled resection of DNA ends that, in turn, trigger ATR-dependent pathways. The latter converge with the former on the master regulator of mitosis, CDK1, blocking its activity.

Entry and transition through mitosis is highly controlled by molecular constrains (checkpoints) that have evolved to prevent genomic instability and consist of the G2/M and the spindle assembly checkpoints. The G2/M checkpoint prevents mitotic entry to cells that have suffered DNA damage during G2 or that have progressed into G2 with unrepaired DNA lesions from previous cell cycle phases. Final target of the G2/M DNA damage checkpoint is CDK1, the master regulator of mitosis. The cascade of phosphorylation events impinging on CDK1 is briefly sketched below. Signals from unfinished DNA replication (through ATR/CHK1), damaged DNA (through ATM/CHK2) or DNA resected at sites of damage (through ATR/CHK1), activate the kinases WEE1/MYT1 that, in turn, phosphorylate T_14_ and Y_15_ in the Gly-rich P-loop of CDK1, causing inhibition of enzymatic activity (Heald et al., 1993; Figure 1). Phosphorylation at these sites does not impair ATP binding, neither sterically nor by electrostatic repulsion (Gould and Nurse, 1989), but rather hampers catalysis (Atherton-Fessler et al., 1993). Additionally, WEE1 enforces the signal of “NO-entry” into mitosis by inactivating CDC25 (Donzelli and Draetta, 2003), the phosphatase responsible for CDK1 dephosphorylation. Specifically, CHK1-dependent phosphorylation of CDC25A at Ser_124_/Thr_507_ and of CDC25C at Ser_216_ mediates interaction with 14-3-3 proteins that, in turn, displace the phosphatases from the nucleus, a mechanism that appears to be the primary way to inhibit the function of these two phosphatases during G_2_ and mitosis (Uto et al., 2004). On the other hand, inhibition of CDC25B, the phosphatase mediating activation of CDK1/Cyclin B at centrosomes during prophase, has been extensively studied in relation to its mitotic role (Gabrielli et al., 1996) but is less characterized in the context of the DNA damage response. Factors upstream of CDC25 or Cyclin B/CDK1, such as the Polo-like kinases PLK1 and PLK3 (Nyberg et al., 2002; Bahassi el et al., 2006), Aurora-A (Ferrari et al., 2005; Krystyniak et al., 2006; Bhatia et al., 2010) and protein phosphatase PP2A (Yan et al., 2010) are also part of the G2/M checkpoint signaling network. Maintenance of the G2/M checkpoint activation partly relies on transcriptional regulation by p53 that induces transcription of the cell-cycle inhibitor p21^CIP1∕WAF1^, and on expressions of 14-3-3s (a scaffold and signaling protein), PUMA (BCL2 binding component 3), BAX (BCL2 partner and apoptotic activator) and GADD45 (growth arrest and DNA-damage-inducible gene) (Nyberg et al., 2002; Riley et al., 2008). Upon completion of DNA synthesis or repair of damage, signals from WEE1 cease, resulting in progressive dephosphorylation and reactivation of CDC25C. The latter, in turn, initiates selective dephosphorylation of the inhibiting sites in CDK1 (Izumi and Maller, 1993), creating an auto-catalytic loop in which CDK1- (Hoffmann et al., 1993; Strausfeld et al., 1994) and Polo-like kinase 1 (PLK1)-dependent CDC25C phosphorylation (Strausfeld et al., 1994; Toyoshima-Morimoto et al., 2002) increase phosphatase activity leading to full dephosphorylation and activation of CDK1. As a result, the checkpoint is silenced and cell cycle progression ensues.

### Spindle assembly checkpoint

The spindle assembly checkpoint (SAC) ensures that chromosomes are properly bi-oriented, preventing missegregation that would otherwise result in aneuploidy (Musacchio and Salmon, 2007). Target of the SAC is the APC/C, an E3 ubiquitin ligase composed of approximately 15 subunits, which binds its substrates by recognizing so-called degron sequences (Pines, 2011). APC/C is activated in mitosis by its co-activators CDC20 and CDH1 in a Cyclin B/CDK1-dependent manner (Wieser and Pines, 2015) and works in tandem with two distinct E2 conjugating enzymes: UBCH5 or UBCH10 that add the first ubiquitin moiety to APC/C substrates, and UBE2S that extends the chain (Rodrigo-Brenni and Morgan, 2007; Garnett et al., 2009) mediating preferentially the formation of K_11_-linked ubiquitin chains (Wu et al., 2010; Bremm and Komander, 2012). K_11_-chains show a distinct fold with respect to K_48_- or K_63_-linked ubiquitin (Matsumoto et al., 2010). A phosphorylation-dependent switch controls timely activation of the E2 UBE2S by the APC/C complex, whereby phosphorylation of Ser_92_ in CDC20 prevents delivery of UBE2S to the APC/C, and its dephosphorylation by PP2A^B56^ allows UBE2S to bind the APC/C, catalyzing ubiquitin chain elongation (Craney et al., 2016).

Major players of the SAC are Mad1, Mad2, Bub1, BubR1/Mad3, Bub3, and Mps1, proteins that essentially monitor kinetochore—microtubule attachments and convert this to signals that inhibit metaphase-to-anaphase transition (Cheeseman, 2014). The main trigger of signals from SAC is Mad2, a protein that can assume an “open” (inactive) or a “close” (active) conformation. Mechanistically, the closed conformation into which Mad2 folds once bound to kinetochores that are improperly attached to spindle microtubules is induced in further neighboring Mad2 molecules that diffuse away from kinetochores and associate with BubR1 and Bub3 forming the so-called mitotic checkpoint complex (MCC). The latter binds and sequesters the first co-activator of APC/C, Cdc20, in an MPS1-dependent manner (Wieser and Pines, 2015), blocking degradation of securin and effectively arresting cells in metaphase (Cheeseman, 2014; Sivakumar and Gorbsky, 2015). The kinases Aurora-B, CDK1 and PLK1 participate in regulating kinetochore function, with Aurora-B-dependent phosphorylation of Ndc80 N-terminus reducing the microtubule-binding affinity of the Ndc80 complex and eliminating incorrect kinetochore-microtubule attachments (Cheeseman et al., 2006). PLK1 associates and regulates several kinetochore proteins, including those localized in the inner centromere like CENP-U, phosphorylation of which facilitates PLK1 recruitment to the kinetochore (Kang et al., 2006), and PLK1-interacting checkpoint helicase (PICH) that binds the kinase through its Polo-box domain (Baumann et al., 2007). Once appropriate attachment is established (i.e., bi-orientation) such that sufficient tension is created and the kinase is spatially separated from its substrates (Liu et al., 2009), PP1 dephosphorylates Aurora-B targets (Cheeseman, 2014), with additional support from PP2A (Foley et al., 2011). Satisfaction of the checkpoint upon appropriate bi-orientation of chromosomes triggers the metaphase to anaphase transition.

## Challenges to the genome and responses in mitosis

In order to preserve the integrity of information contained in the genome, DNA is continuously monitored by proteins that recognize distinct types of damage. Such proteins or protein complexes—so called sensors—inform signal transducers that, in turn, prompt effectors to orchestrate repair of the damage (Bologna and Ferrari, 2013; Jackson and Durocher, 2013; Dantuma and van Attikum, 2016). In parallel, transducers trigger checkpoint pathways impinging on key cell cycle controllers (see above) that ultimately slow down or arrests transition through the cell cycle (Kastan and Bartek, 2004). Inappropriate detection or untimely repair of DNA damage before the onset of mitosis may lead to chromosome breaks, rearrangements or fusions—comprehensively know as “structural abnormalities”—that facilitate the development of cancer (Branzei and Foiani, 2010; Curtin, 2012) and have been focus of intense research in the last decades (Aguilera and García-Muse, 2013).

DNA repair involves chromatin remodeling that, in turn, facilitates binding of repair factors to the region(s) where the lesion occurred (Aydin et al., 2014). This sequence of events has been observed during transition through the cell cycle, when the DNA repair machinery called to action faces simple or more challenging tasks, depending on whether damage is in euchromatin or in heterochromatin (Lemaître and Soutoglou, 2014). It appears, however, that DNA damage responses operative till completion of G2 and mediated through checkpoint kinases converging on WEE1 and CDK1 (Boddy et al., 1998; Smith et al., 2010; Figure 1), must be blocked at the time of chromosome condensation and segregation. In cells carrying a wild type complement of checkpoint genes, entry into prometaphase with ensuing chromosome condensation and nuclear envelope breakdown defines a point of non-return and puts an end to the checkpoint that was operative in G2.

**Figure 2:**
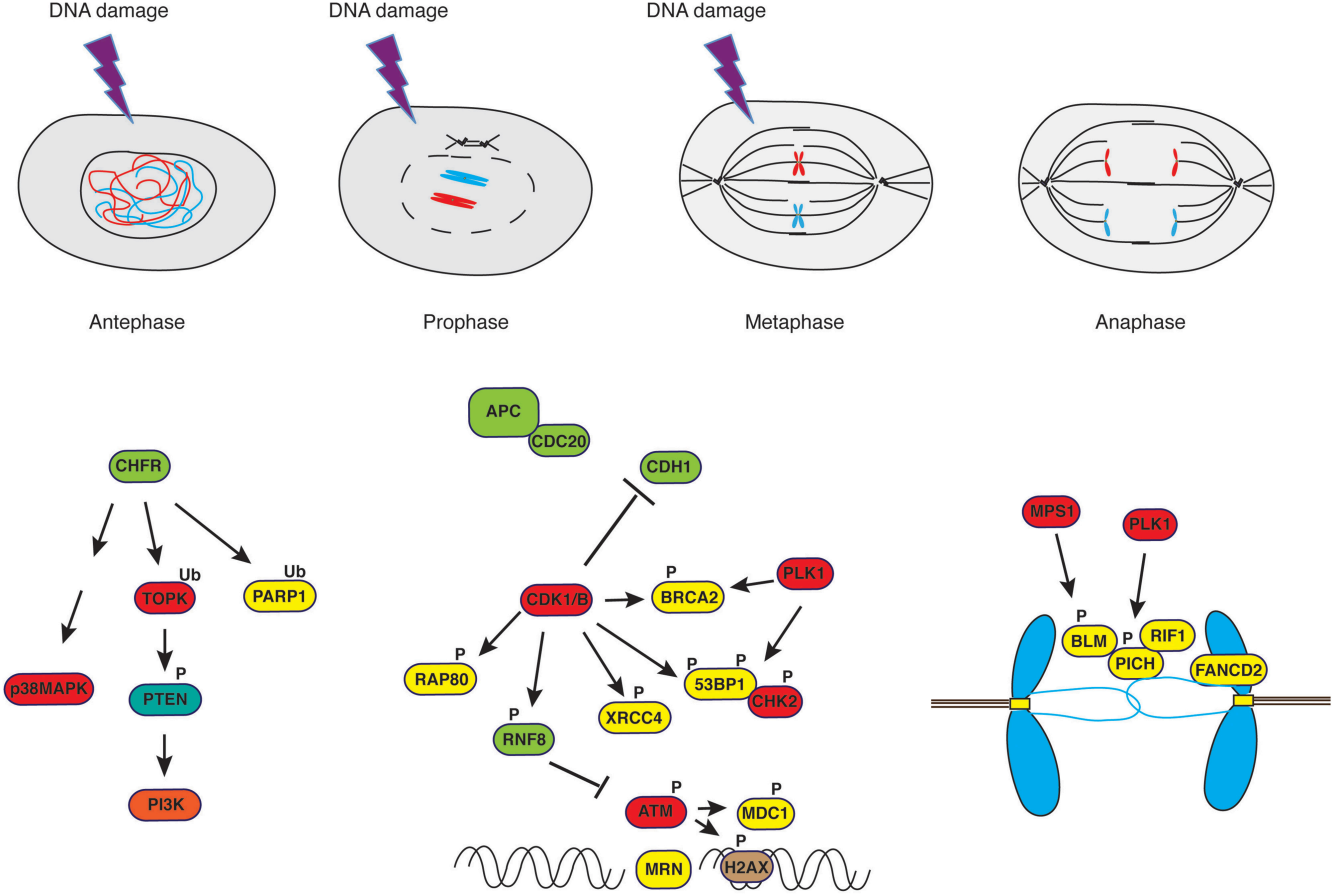
**DNA damage response in mitosis**. Irradiation of cells in antephase or in early prophase triggers a response that is independent of PI-3K-like kinases such as ATM but rather depends on the E3-ubiquitin ligase CHFR and the stress-response kinase p38MAPK. On the other hand, irradiation of cells in late prophase or in metaphase leads to a curtailed DNA damage response. Ultrafine anaphase bridges, caused by improper resolution of replication or recombination intermediates, are addressed by the coordinated action of the helicases PICH and BLM supported by RIF1 (see text for details).

Termination of activities on DNA in mitosis is exemplified by the repression of transcriptional activity (Martínez-Bálbas et al., 1995) that occurs through a passive process, consisting in limited access of transcription machinery to compacted chromatin, and an active mechanism, entailing CDK1-dependent phosphorylation of its components (Gottesfeld and Forbes, 1997). Similar mechanisms control DNA repair proteins, to avoid that active DSB repair during mitosis may result in telomere fusions, aneuploidy (Cesare, 2014; Orthwein et al., 2014) and whole chromosome missegregation through collateral stabilization of kinetochore-microtubules interactions (Bakhoum et al., 2014). Indeed, it was observed that in the absence of genotoxic stress, DNA repair proteins are phosphorylated in mitosis in a CDK- or PLK1-dependent manner to exclude them from chromatin (Figure 2). This is the case for BRCA2 (Lee et al., 2004), RAP80 (Cho et al., 2013), 53BP1 (Orthwein et al., 2014; Benada et al., 2015), RNF8 (Orthwein et al., 2014) and XRCC4 (Terasawa et al., 2014), to mention just few examples. RNF8, a well-characterized E3 ubiquitin ligase recruited to sites of damage through interaction of its N-terminal FHA domain with phosphorylated MDC1 and HERC2 (Bologna and Ferrari, 2013), the latter acting as coordinator of ubiquitin-dependent assembly of DNA repair factors (Bekker-Jensen et al., 2010), is phosphorylated by mitotic kinases to suppress its interaction with MDC1 (Orthwein et al., 2014). In the case of 53BP1, phosphorylation of two residues within the ubiquitylation-dependent recruitment (UDR) motif of 53BP1 in mitosis blocks binding to K_15_-ubiquitylated histone H2A, thus impairing its recruitment to foci (Benada et al., 2015). On the other hand, PP4C/R3β-mediated dephosphorylation of these sites in G1 re-establishes 53BP1 binding to chromatin (Lee et al., 2014). In the case of BRCA2, PLK1-dependent phosphorylation at S_193, 205, 206_ and T_203, 207_ causes dissociation from the histone acetyltransferase protein p300/CBP-associated factor (P/CAF) (Lin et al., 2003), and CDK-dependent S_3291_ phosphorylation at the onset of mitosis inhibits BRCA2-mediated stabilization of RAD51 nucleofilaments that are normally generated at sites of recombination (Esashi et al., 2005).

Shutting off repair in mitosis, however, does not imply that DNA damage is ignored if it occurs in this phase of the cell cycle. Evidence obtained in early studies conducted in vertebrate somatic cells showed that chromosome fragmentation caused by irradiation at the beginning of mitosis persisted till anaphase (Zirkle and Bloom, 1953), possibly indicating that repair pathways were not activated in this period of time. On the other hand, recent studies on the outcome of laser irradiation of mitotic chromosomes indicated that DNA damage response is triggered within 30 s from the treatment (Gomez-Godinez et al., 2010). Studies in the budding yeast *S. cerevisiae*, where activation of a dicentric chromosome was used to introduce a double strand DNA break into a chromosome at mitosis, showed that cells paused in mid-anaphase, triggering RAD9-dependent events that were reminiscent of a DNA damage response (Yang et al., 1997). Subsequent work conducted in yeast, where cells were irradiated in mitosis, showed that stabilization of Pds1, an anaphase inhibitor and APC/Cdc20 target, led to delay of anaphase and mitotic exit, facilitating repair of damage (Tinker-Kulberg and Morgan, 1999). Delay of the metaphase-to-anaphase transition was also reported for *Drosophila* embryos undergoing irradiation or being treated with methly metane sulfonate (MMS) and it was shown to depend on the stabilization of Cyclin A (Su and Jaklevic, 2001). Mitotic DNA damage in *X. laevis* and DT40 cells was shown to prevent spindle assembly in an ATM/ATR-dependent manner (Smith et al., 2009), and proposed to be an additional means to monitor chromosome breaks that have escaped the G2/M checkpoint.

It has been reported that eukaryotic cells are able to delay the execution of mitosis or, in some instances, reverse progression through mitosis, in response to DNA damage (Rieder and Cole, 1998) or microtubule poisons (Rieder and Cole, 2000) administered in antephase, a time when microscopic changes in the cell are not yet detectable and that physically spans from the conclusion of G2 to the initiation of chromosome condensation (Chin and Yeong, 2010). The same response was observed upon damage caused in early prophase (Rieder and Cole, 1998). In antephase, cells activate a checkpoint that is not mediated by PI-3K-like kinases such as ATM but rather depends on two proteins, the CHFR E3-ubiquitin ligase (Matsusaka and Pines, 2004; Shinde et al., 2013) that principally catalyzes poly-ubiquitylation of its substrates via K_48_ and K_63_ (Kang et al., 2002; Bothos et al., 2003; Oh et al., 2009) and is involved in the first wave of ubiquitylation at DNA damage sites (Liu et al., 2013a), and the Pro-directed p38 MAPK (Mikhailov et al., 2004; Figure 2). Cells containing a wild-type antephase checkpoint undergo chromosome decondensation and revert to a G2-like state (Rieder and Cole, 1998; Matsusaka and Pines, 2004), whereas cells lacking a functional CHFR progress into mitosis (Scolnick and Halazonetis, 2000). Extensive damage occurring upon completion of antephase does not normally cause reversion to an early stage of the cell cycle but rather triggers mitotic arrest through activation of SAC (Mikhailov et al., 2002, 2004; Choi and Lee, 2008), the only mechanism left in the arsenal of cells at this point of the cell cycle. SAC, however, does not orchestrate repair of damaged DNA but monitors that distribution of chromosomes to daughter cells occurs equally, hence avoiding aneuploidy. As mentioned above, SAC is active at kinetochores where the state of microtubule attachment is monitored, and signaling pathways preventing anaphase remain active as long as mono-oriented or incorrectly attached kinetochores are detected (Mikhailov et al., 2002; Cheeseman and Desai, 2008). Observations made in yeast (Pangilinan and Spencer, 1996) and in mammalian cells (Mikhailov et al., 2002) indicate that altering the topology of chromatin, particularly at regions that affect kinetochore structure, prevents satisfaction of SAC and delays transition through mitosis. These studies showed that ATM-dependent pathways (Mikhailov et al., 2002) or DDR genes (Pangilinan and Spencer, 1996) are not involved in the response to chromosome damage and that the metaphase block can be rapidly overridden by dominant-negative Mad2 (Mikhailov et al., 2002). However, a study addressing the effect of decatenation inhibitors (topoisomerase-II inhibitors) on nocodazole-arrested cells described a number of ATM-dependent events in response to these drugs, including H2AX phosphorylation, CDK1 inactivation, histone H3 dephosphorylation and chromosome decondensation, paralleled by stabilization of Cyclins A and B1, with cells apparently unable to exit mitosis (Chow et al., 2003). Studies conducted in our laboratory on cells that were synchronized in mitosis without disturbing microtubule dynamics, hence without “pre-sensitizing” cells by activation of stress responses that are known to increase levels of γH2AX (Giunta et al., 2010), and that we treated with ionizing radiation at metaphase, showed CHK2 activation, rapid inhibition of CDK1 and Aurora-A activities accompanied by reactivation of PP1, increased APC/CDH1 E3-ubiquitin ligase activity and chromosome decondensation (Bhatia et al., 2010). Our data clearly showed that metaphase-irradiated cells completed mitosis at the expenses of genome stability, displaying increased chromosome segregation defects and the formation of micronuclei (Bhatia et al., 2010).

A comprehensive study that assessed entity and amplitude of the DDR in mitosis by scoring formation of IR-induced foci (IRIF) and comparing mitotic to interphase cells concluded that only a subset of IRIF could form in mitosis, namely those comprising γ-H2AX, NBS1 and MDC1, but not RNF8, RNF168, BRCA1 or 53BP1 (Giunta et al., 2010) as also confirmed by others (Nelson et al., 2009). Exclusion of RNF8 and 53BP1 from chromatin was shown to be the consequence of phosphorylation by mitotic kinases (see above) (Orthwein et al., 2014) and association of 53BP1 to IRIF was observed only upon nuclear envelope reformation around decompacting chromosomes in telophase (Giunta et al., 2010; Figure 2).

Specifically regarding ATM, its activation has been examined in mammalian cells both during undisturbed transition through mitosis or upon stress. In the absence of DNA damage, the kinase Aurora-B phosphorylates ATM on S_1403_ in mitosis, and abrogation of this event was shown to impair signaling through the spindle assembly checkpoint (Yang et al., 2011). Administration of taxol, a drug suppressing microtubule dynamics and causing mitotic stress, was reported to trigger ATM activity, though none of the known ATM targets in DDR such as SMC-1, NBS-1 or CHK-2 was phosphorylated under these conditions (Shen et al., 2006). DNA damage response-related roles for ATM in mitosis were inferred from early observations made in lymphoblastoid cells derived from A-T patients, which displayed a defective SAC upon treatment with radiation (Takagi et al., 1998; Shigeta et al., 1999). Another report described the activation of ATM in response to chromosomal breaks generated during mitotic catastrophe (Imreh et al., 2011). ATM activation was also examined upon irradiation of cells synchronized in mitosis with drugs that interfere with microtubule polymerization. Under these conditions, ionizing radiation triggered ATM activity, though CHK2 failed to fire and cells remained in mitosis with elevated phosphorylation at MPM-2 epitopes, indicative of high CDK1 activity. Mechanistically, the absence of a productive DDR signal following ATM activation was proposed to result from PLK1-dependent phosphorylation of CHK2, with 53BP1 acting as platform to bring PLK1 and CHK2 in close proximity (van Vugt et al., 2010; Figure 2).

As a whole, these studies confirm that ATM can fire when the minimal requirement for its activation is satisfied, namely the presence of exposed double-stranded ends (You et al., 2007), independently on the cell cycle position, though a productive DDR downstream of ATM seems not to be triggered in early mitosis.

In addition to DNA damage occurring during transition through mitosis, cells reaching mitosis are confronted with other problems: these are the structures resulting from incomplete DNA replication, improper resolution of replication intermediates or unresolved intermediates of homology-directed repair carried over from S-phase (Liu et al., 2014). Such structures become a threat at the time of chromosome segregation since they can cause sister chromatid entanglement and non-disjunction (Gelot et al., 2015). Incomplete DNA replication occurs at regions encompassing so-called “replication barriers.” Predominant among those are common-fragile sites (CFSs) (Durkin and Glover, 2007), cytologically defined as segments in metaphase chromosomes displaying brakes at runs of flexible AT-rich repeats (Aguilera and García-Muse, 2013). CFSs constitute up to 80% of the breakpoints that lead to the gross chromosomal rearrangements (GCRs) observed in precancerous cells (Bartkova et al., 2006). Part of under-replicated CFSs observed in cells at anaphase remain connected through thin threads of DNA called ultrafine bridges (UFBs) (Liu et al., 2014). To avoid DNA breaks resulting from segregation of incompletely replicated chromosomes, these structures are addressed before cell division. It has been observed that BLM, along with topoisomerase IIIα, RMI1, RMI2 (BTRR complex) and PICH (PLK1-Interacting Checkpoint Helicase), coat anaphase UFBs (Baumann et al., 2007; Chan and Hickson, 2009; Chan et al., 2009; Naim and Rosselli, 2009; Figure 2). An earlier report on BLM phosphorylation by MPS1, facilitating accurate chromosome segregation (Leng et al., 2006), anticipated the important role played by this DNA helicase in mitosis. BLM recruitment to UBFs is facilitated by FANCD2, a key component of the Fanconi Anemia pathway, which was shown to form sister *foci* in mitosis (Naim and Rosselli, 2009; Harrigan et al., 2011; Lukas et al., 2011; Figure 2) and be necessary to prevent the generation of micronuclei (Naim and Rosselli, 2009). The SNF2 ATPase family member PICH plays an essential role at kinetochores and the inner centromere, as demonstrated by studies in which PICH depletion caused loss of Mad2 from kinetochores and abrogated the spindle checkpoint, events that were followed by chromosome missegregation (Baumann et al., 2007). Also PICH was proposed to help recruiting the BTRR complex at UFBs, cooperating to the resolution of DNA bridges by the end of anaphase (Liu et al., 2014; Figure 2). A recent addition to the pool of proteins present at UFBs is RIF1, ortholog of a yeast telomeric protein. RIF1 is recruited to UFBs in a PICH-dependent manner but independently of 53BP1, ATM or BLM, and phosphorylation by CDK1 restricts its ability to bind DNA at anaphase (Hengeveld et al., 2015). In addition to the BTRR complex, the Holliday Junction resolvases SLX1–SLX4–MUS81–EME1 (SLX–MUS complex) and GEN1 (Wyatt et al., 2013; Chan and West, 2014) contribute to process structures caused by under-replication at CFSs (Naim et al., 2013; Ying et al., 2013). The SLX–MUS complex cooperates with TopBP1, a scaffold protein composed of nine BRCT domains and recruited at sites of DNA damage in a 9-1-1-dependent manner (Delacroix et al., 2007; Lee et al., 2007; Wardlaw et al., 2014). TopBP1 is necessary for ATR activation (Kumagai et al., 2006), colocalizes with RPA and FANCD2 (Pedersen et al., 2015) forming *foci* on condensing chromatin through its BRCT5 domain, and recruits TOP2A to help resolving DNA entanglements between sister chromatids (Broderick et al., 2015). CDK1-dependent phosphorylation of EME1 in the MUS81-EME1 structure-specific endonuclease, promoting interaction with SLX1-SLX4, controls the resolution of DNA recombination intermediates in mitosis (Matos et al., 2011; Matos and West, 2014). Proteome-wide studies have identified a number of ubiquitylation sites in GEN1, MUS81, EME1, TopBP1 (Kim et al., 2011; Wagner et al., 2011; Mertins et al., 2013), though the biological function of such PTM and its eventual connection with mitotic functions of these proteins has not been addressed to date. Finally, human GEN1 acts as back up to the above-mentioned machinery at anaphase, moving in place and gaining access to DNA after nuclear envelope breakdown (NEB) (Wechsler et al., 2011; Chan and West, 2014; Sarbajna et al., 2014). For the yeast homolog of GEN1, Yen1, it was shown that activity and access to the nucleus depend on a reversible CDK1/Cdc14 phosphorylation switch (Eissler et al., 2014; Matos and West, 2014).

In addition to the role of the above mentioned scaffold proteins in tethering nucleases to UFBs to the end of resolving DNA bridges in anaphase, unscheduled DNA synthesis at UFBs marked by TopBP1 (Pedersen et al., 2015) or SLX4 (Minocherhomji et al., 2015) has been reported and interpreted as an attempt to fill-in unreplicated regions, hence restoring genome integrity before cell division.

In case lesions generated by replication stress remain unrepaired, they are passed to daughter cells in a manner that shelters them from further damage through sequestration in 53BP1 nuclear bodies, thus allowing repair in the next cell cycle (Lukas et al., 2011). In the presence of extensive damage that remains unaddressed, cells experience sudden mitotic death also known as mitotic catastrophe (Morrison and Rieder, 2004; Vitale et al., 2011).

We have mentioned above that the DNA damage checkpoint is in place to facilitate DNA repair by blocking transition from G2 to M (Figure 1). A non-trivial consequence of prolonged arrest before mitosis is centrosome amplification, an event that is observed with high incidence in cancer cells carrying mutations of DNA repair genes. This event, which is alleviated upon bypass of the checkpoint in a manner that is only partially dependent on ATM, was postulated to be a mechanism ensuring death of cells that manage to evade the G2/M checkpoint or the SAC (Dodson et al., 2004). The metaphase-to-anaphase transition is a critical cell cycle stage during which chromosome missegregation may occur. Loss or gain of entire chromosomes—known as “numerical abnormalities”—resulting from chromosome missegregation during mitosis, is a characteristic of tumors known from more than a century and described as “aneuploidy” (Pellman, 2007). Mechanisms leading to aneuploidy have been amply reviewed elsewhere (Holland and Cleveland, 2009) and comprise (i) defective attachment of sister chromatids to spindle microtubules (merotelic attachment), often linked to centrosome amplification, (ii) malfunction of the spindle assembly checkpoint and (iii) defects in chromosome cohesion.

Key to a fully-fledged response to DNA damage is the network of signals that orchestrate assembly of DNA repair proteins at sites of damage and informs the cell cycle machinery. Ultimate target of G2/M checkpoint pathways is CDK1, the master regulator of mitosis (see “Mitosis and Checkpoints”) that is maintained in an “OFF” status by direct negative inputs (WEE1) and inactivation of its positive regulators (CDC25), in conjunction with modulation of other enzymatic activities such as those of the kinases PLK1 (Smits et al., 2000), Aurora-A (Krystyniak et al., 2006) and protein phosphatase PP2A (Yan et al., 2010; Figure 1). The budding yeast *S. cerevisiae* represents a notable exception in this respect. Whereas in high eukaryotes CDKs have acquired specific functions throughout evolution, with CDK1 being the master controller of mitosis and undergoing immediate inhibition in an ATM/ATR-CHK1/CHK2-dependent manner upon DNA damage, *S. cerevisiae* possesses only one Cyclin-dependent kinase, Cdc28, controlling pathways and transitions in all phases of the cell cycle and whose activity depends on interaction with different Cyclins (Enserink and Kolodner, 2010). As opposed to CDK1, budding yeast Cdc28 is not inhibited by DNA damage response pathways, since the status of Tyr_19_ phosphorylation in the P-loop of Cdc28 is not a determinant for entry into mitosis (Amon et al., 1992). The key control of budding yeast mitosis is operative at the metaphase-to-anaphase transition, where degradation of the Esp1 (separase) inhibitor Pds1 (securin) allows cleavage of the Scc1 subunit in the cohesin complex and separation of the sisters (Ciosk et al., 1998; Sanchez et al., 1999). Hence, in yeast, mitotic arrest in response to DNA damage occurs in metaphase and depends on the abundance of Pds1 (Sanchez et al., 1999). This mechanism liberates Cdc28 of the control that CDK1 undergoes to in higher eukaryotes. Contrary to rapid inhibition upon DNA damage, Cdc28 is absolutely required in DDR and participates to the control of genome stability (Enserink et al., 2009). Cdc28 triggers homologous directed repair of DSBs through phosphorylation of Sae2 (Huertas et al., 2008), prompting initial resection of DNA ends (Ira et al., 2004), and other components of error-free repair pathways such as Dna2 (Ubersax et al., 2003) and Srs2 (Chiolo et al., 2005; Saponaro et al., 2010). Interestingly, Cdc28 targets such as Sae2 are also phosphorylated by classic DDR kinases, whereby mutation of phosphorylation sites for either set of kinases hampers repair and recombination functions of the protein (Baroni et al., 2004).

Hence, the rapid inactivation of vertebrate CDK1 in response to damage is difficult to reconcile with claims on its involvement in DNA damage responses at G2/M. Although it has been suggested that the gap between checkpoint triggering and CDK1 shutoff in vertebrate cells may be sufficient for CDK1 to orchestrate initial phases of repair, a much wiser interpretation of the experimental evidence is that repair of DNA damage in checkpoint-arrested cells depends on CDK2 (Wohlbold and Fisher, 2009) and other Proline-directed kinases involved in stress responses (Bulavin et al., 2001). As a matter of fact, high CDK1 activity, along with the activity of other mitotic kinases (Benada et al., 2015), is sufficient to suppress responses to DNA damage occurring during transition through mitosis in mammalian cells (Zhang et al., 2011). A further layer of regulation is imposed by phosphatases such as WIP1, a CDK1 target that undergoes ubiquitin-mediated degradation in mitosis, which sets the threshold for DDR signaling in mitosis by controlling the phosphorylation state of DDR proteins (Macurek et al., 2013).

## Mitotic PTMs and cancer therapy

Mitosis is the cell cycle phase that is most vulnerable to injury, regardless on whether damage is caused by radiation, heat-shock or chemicals (Westra and Dewey, 1971; Stobbe et al., 2002; Chan et al., 2012). Based on this indication, targeting mitotic cells has been largely exploited in the clinic as means to contain tumor growth (Doménech and Malumbres, 2013; Marzo and Naval, 2013). Molecular studies have highlighted the role of PTMs, and the enzymes that mediate them, in mechanisms controlling the mitotic responses to stress (Pearce and Humphrey, 2001). This comes as no surprise, considering that essentially all mechanistic aspects of normal transition through mitosis are controlled by PTMs of mitotic machinery components, with reversible PTMs allowing a certain degree of flexibility in the decisions implemented and irreversible PTMs conferring directionality to the process (Nigg, 2001; Ma and Poon, 2011; Teixeira and Reed, 2013). Hence, mitotic protein kinases and E3-ubiquitin ligases with established role in cancer have become the focus of interest for chemists and pharmacologists designing and testing novel therapeutics that target cells in mitosis (Dominguez-Brauer et al., 2015). Such interest was also motivated by considerations on the side effects of classic anti-mitotic drugs like taxanes and vinca alkaloids that are currently deployed to the treatment of a variety of solid tumors such as breast, ovarian and lung cancer. Anti-mitotic drugs, due to their mode of action that alters microtubules' dynamic instability, result in neurotoxicity and neutropenia (Marzo and Naval, 2013). Furthermore, their lack of efficiency when used as single agents has evidenced another important limitation of these anti-mitotics (Doménech and Malumbres, 2013; Marzo and Naval, 2013). Shifting the focus to the discovery of drugs that target mitotic kinases or E3-ubiquitin ligases, however, did not solve the major caveat for cell cycle—and mitotic—inhibitors, namely the fact that the efficacy of a drug depends on the tumor proliferative rate: fast proliferation makes leukemia and myeloma relatively favorable conditions to treat, whereas a mitotic index (i.e., the percentage of mitotic cells in the whole populations) as little as 1% and doubling time of more than 1 year, as observed in some solid tumors, are negative factors to be taken into account when planning a treatment and its length.

**Figure 3:**
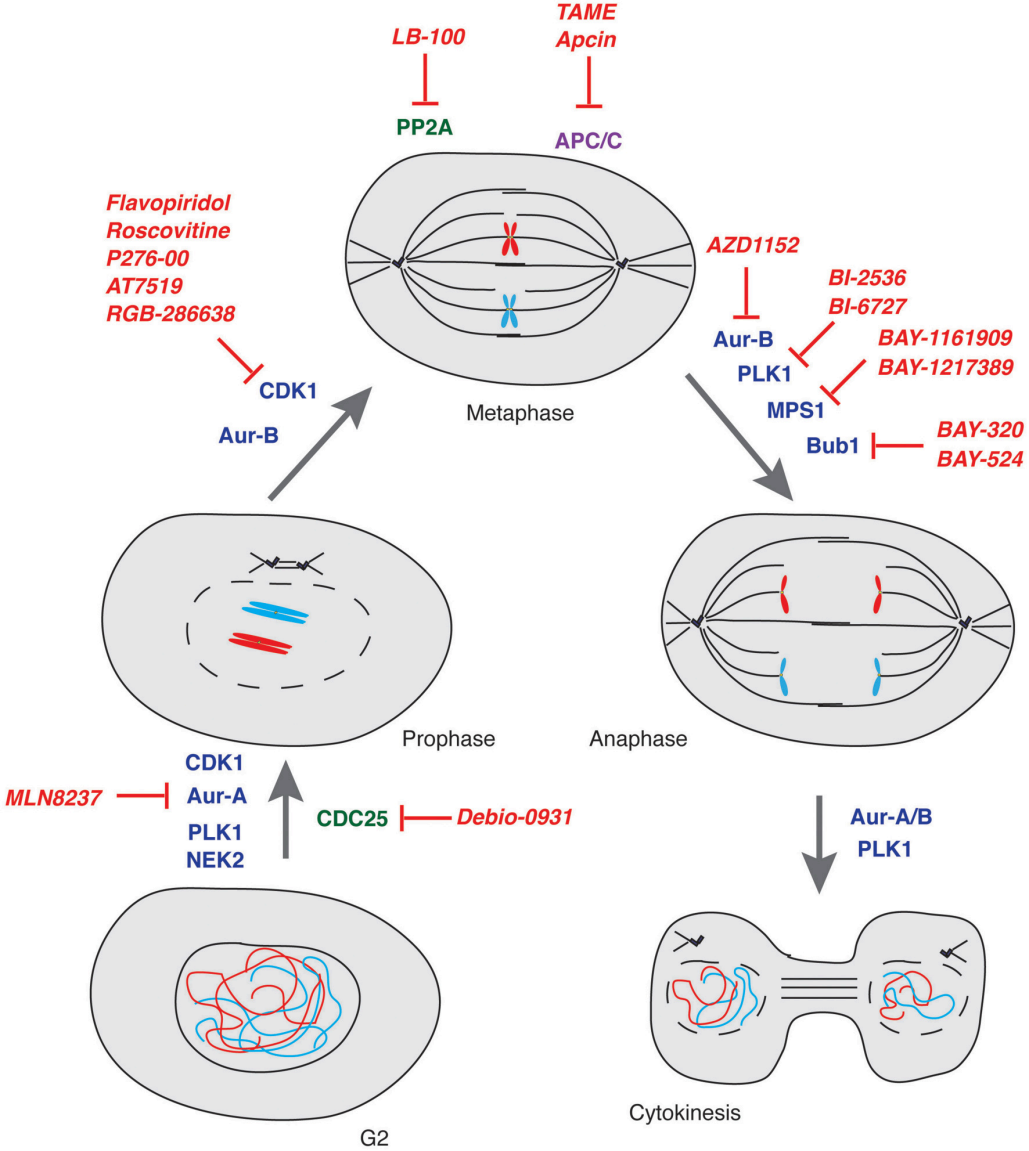
**Mitosis and its control by kinases, phosphatases and E3-ubiquitin ligases**. Schematic representation of key controllers of the onset, transition and exit from mitosis with indication of the major drugs inhibiting their function. Kinases: blue; Phosphatases: green; E3-ubiquitin ligases: purple; Drugs: red.

Here below we provide a report on the current status of drug discovery and clinical trials for compounds targeting mitotic kinases and phosphatases as well as ubiquitin-proteasome system components (Table 1 and Figure 3).

**Table 1:** **List of drugs, their mitotic targets and current clinical trial phase**.

**Drug**	**Target**	**Status**	**References**
**MITOTIC KINASE INHIBITORS**			
Roscovitine (Cyclacel)	CDK2, CDK7, CDK9	Phase I-II	De Azevedo et al., 1997
AT7519 (Astex)	pan-CDKs	Phase I-II	Wyatt et al., 2008
Dinaciclib (Merck)	pan-CDKs	Phase I-II-III	Parry et al., 2010
Flavopiridol (Sanofi-Aventis)	pan-CDKs	Phase I-II	De Azevedo et al., 1996
P276-00 (Piramal)	pan-CDKs	Phase I-II	Joshi et al., 2007
RGB 286638 (Agennix)	pan-CDKs and others	Phase I-II	Cirstea et al., 2013
Terameprocol (Erimos)	CDK1 and Survivin	Phase I-II	Heller et al., 2001; Chang et al., 2004
TG02 (Tragara)	pan-CDKs, JAK2, FLT3	Phase I	Goh et al., 2012
MK-1775 (Merk)	Wee1	Phase I-II	Hirai et al., 2009
BI-2536 (Boehringer Ingelheim)	Plk1	Phase I-II	Lenart et al., 2007
Volasertib/BI-6727 (Boehringer Ingelheim)	Plk1	Phase I-II-III	Rudolph et al., 2009
CFI-400945 (Campbell Family Institute, CAN)	Plk4	Phase I	Mason et al., 2014
AMG-900 (Amgen)	Aurora-kinases	Phase I	Payton et al., 2010
AT-9283 (Astex)	Aurora-kinases	Phase I-II	Howard et al., 2009
CYC-116 (Cyclacel)	Aurora-kinases	Phase I	Wang et al., 2010
PHA-680632 (Pfizer/Nerviano MS)	Aurora-kinases	Phase II-III	Soncini et al., 2006
GSK1070916 (GlaxoSmithKline)	Aurora-kinases	Phase I	Hardwicke et al., 2009
PF-03814735 (Pfizer)	Aurora-kinases	Phase I	Jani et al., 2010
Danusertib/PHA-739358 (Pfizer/Nerviano MS)	Aurora-kinases	Phase II	Carpinelli et al., 2007
R763/AS703569 (Rigel)	Aurora-kinases	Pre-Clinical	McLaughlin et al., 2010
SNS-314 (Sunesis)	Aurora-kinases	Phase I	Oslob et al., 2008
MK-0457 (VX-680) (Vertex/Merck) Tozasertib	Aurora-kinases	Phase I-II	Harrington et al., 2004
ENMD-2076 (EntreMed)	Aurora-A	Phase I-II	Tentler et al., 2010
Alisertib/MLN8237 (Millennium)	Aurora-A	Phase I-II	Görgün et al., 2010
Barasertib/AZD1152 (AstraZeneca)	Aurora B	Phase I-II-III	Mortlock et al., 2007; Wilkinson et al., 2007
2OH-BNPP1	Bub1	Pre-Clinical	Kang et al., 2008; Nyati et al., 2015
BAY-320/BAY-524 (Bayer)	Bub1	Pre-Clinical	Baron et al., 2016
Cycloalkenepyrazoles	Bub1	Pre-Clinical	Brazeau and Rosse, 2014
BAY 1161909/BAY 1217389 (Bayer)	Mps1	Phase I	Wengner et al., 2016
CFI-402257 (Campbell Family Institute, CAN)	Mps1	Pre-Clinical	Dominguez-Brauer et al., 2015
S81694 (Nerviano MS)	Mps1	Pre-Clinical	Colombo et al., 2015
CRT0105446	LIMK1 and LIMK2	Pre-Clinical	Mardilovich et al., 2015
CRT0105950	LIMK1 and LIMK2	Pre-Clinical	Mardilovich et al., 2015
**MITOTIC PHOSPHATASE INHIBITORS**			
IRC 083864/Debio 0931 (Ipsen -DebioPharma)	CDC25	Phase II (^*^)	Lavecchia et al., 2010
LB100 (Lixte biotechnology)	PP2A	Pre-clinical/Phase I	Lu et al., 2009
**UBIQUITIN-PROTEASOME SYSTEM INHIBITORS**			
Bortezomib (Millennium)	Proteasome	Phase I-II	Hideshima et al., 2001
Carfilzomib (Onyx Pharmaceuticals)	Proteasome	Phase I-II	Kortuem and Stewart, 2013
MLN9708 (Millennium)	Proteasome	Phase I-II	Chauhan et al., 2011
CEP-18770 (Cephalon)	Proteasome	Phase I-II	Seavey et al., 2012
TAK-243 (MLN7243, Millennium - Takeda)	E1 (UBA1)	Pre-clinical/Phase I	Milhollen et al., 2015
Nutlins (Roche)	E3 (MDM2)	Pre-clinical	Vassilev, 2007
TAME	E3 (APC/C - Cdc20)	Pre-clinical	Zeng et al., 2010
Apcin (Harvard U - Boston Biochem)	E3 (APC/C - Cdc20)	Pre-clinical	Sackton et al., 2014
MLN4924 (Millennium)	NEDD8 activating enzyme (NAE)	Phase I-II	Soucy et al., 2009

* *Since launch in Phase II, no additional information has been rendered available at ClinicalTrials.gov*.

### Inhibition of mitotic kinases

#### CDK1 inhibitors

CDK1 is the master regulator of mitosis (Nigg, 2001). Flavopiridol is the first CDK1 inhibitor that underwent >60 clinical trials to date (www.clinicaltrials.gov). The poor efficacy of the compound, however, prevented its approval as anti-tumor drug (Shapiro, 2006; Stone et al., 2012; Galons et al., 2013). Other CDK1 inhibitors displaying high potency on cancer cell lines are currently in Phase I or II (with none of them being yet available for patients, see Table 1 and references therein). So far, only the CDK1 inhibitor Dinaciclib was tested in a phase III study that was concluded in 2015 and aimed at treating refractory chronic lymphocytic leukemia patients (NCT01580228). In general, however, inhibition of CDK1 in healthy cells and the poor selectivity of CDK1 inhibitors, possibly due to the high degree of sequence conservation in the catalytic domain of CDK members, often result in major side effects when deployed in the clinic. For such reason, CDK1 inhibitors are currently used in combination therapies with other mitotic inhibitors (see below).

#### Aurora kinases inhibitors

Aurora kinase family members, Aurora-A, -B and -C exert different roles in the cell. Early studies showed that Aurora-A controls centrosomes maturation and separation, bipolar spindle formation and chromosomes segregation, while Aurora-B, as member of the Chromosome Passenger Complex, participates in the control of chromosome condensation and orientation on the mitotic spindle, ensuring correct kinetochore-microtubule attachments (Nigg, 2001). Both Aurora-A and -B were shown to stabilize midzone microtubules and regulate cytokinesis (Carmena et al., 2009). Aurora-C has a role in gametogenesis, it is expressed in testis, thyroid, and placenta and its contribution to cancer development was shown in mouse models (Khan et al., 2011). Several inhibitors against Aurora-A and Aurora-B have been developed during the last decade (Doménech and Malumbres, 2013; Bavetsias and Linardopoulos, 2015; D'Assoro et al., 2015; Falchook et al., 2015). As for CDKs, most Aurora kinases inhibitors target all family members and a major effort has been done to develop drugs that are more selective for individual Aurora kinases (Table 1 and Figure 3). Among them, two reversible ATP competitive inhibitors, MLN8054 (Manfredi et al., 2007) and its derivative MLN8237 (Manfredi et al., 2011) have shown to be potent and selective Aurora-A inhibitors (Sells et al., 2015). Both were deployed in several studies and since MLN8237 has shown to be safer, it is currently under evaluation in Phase III clinical trials (NCT01482962).

#### Polo-like kinases inhibitors

Polo-like family members constitute another class of Serine/Threonine (Ser/Thr) kinases with key roles in mitosis (Nigg, 2001). Five PLKs are expressed in human cells, PLK1-5, with PLK1 and PLK4 being the major representatives of this family (Zitouni et al., 2014). Distinguishing feature of PLKs is the polo-box domain that flanks the catalytic domain and allows docking to substrates primed by CDKs to carry on their phosphorylation (Zitouni et al., 2014).

Mechanistically, PLK1 is activated by Aurora-A (Ferrari et al., 2005; Macurek et al., 2008) at the onset of mitosis and functions to promote centrosome maturation and separation, assembly and elongation of the mitotic spindle as well as cytokinesis (Barr et al., 2004; Degenhardt and Lampkin, 2010). PLK1 is overexpressed in various malignancies (Holtrich et al., 1994; Eckerdt et al., 2005; Mito et al., 2005; Takai et al., 2005; Strebhardt and Ullrich, 2006; Renner et al., 2009; Weiß and Efferth, 2012). PLK1 inhibition in cancer patients has been pursued with some success using two ATP-competitive kinase inhibitors: BI-2536 and BI-6727 (see Table 1). The potency, pharmacokinetic and pharmacodynamic properties of BI-6727 as well as its antitumor activity in a number of cancer models (Rudolph et al., 2009) has promoted the drug to a phase III trial for acute myeloid leukemia patients where BI-6727 was tested in combination with the DNA-synthesis blocking agent cytarabine (NCT01721876).

PLK4 has a fundamental role in centriole duplication (Bettencourt-Dias et al., 2005; Habedanck et al., 2005). PLK4 overexpression leads to the formation of extra centrosomes resulting in aberrant mitotic spindles and aneuploid daughter cells (Basto et al., 2008; Ganem et al., 2009; Holland et al., 2010). Evidence on PLK4 overexpression in tumors (Macmillan et al., 2001; van de Vijver et al., 2002; Miller et al., 2005; Hu et al., 2006; Salvatore et al., 2007; Chng et al., 2008) raised the interest to develop small molecule inhibitors of this kinase. CFI-400945 was shown to be a potent and selective PLK4 inhibitor exerting a dose-dependent effect on centriole biogenesis (Mason et al., 2014). At high concentration, CFI-400945 inhibits centriole duplication, while at low concentration it causes the generation of supernumerary centrosomes. Interestingly, in both cases, cells arrest or die (Mason et al., 2014). In the same study, the anti-cancer potential of CFI-400945 was also shown in mice and the drug is currently under evaluation in advanced cancer patients (NCT01954316).

Supernumerary centrosomes occur at high frequency in cancer cells but not in non-transformed cells and were originally proposed by Theodor Boveri to be linked to cancer development (Brinkley and Goepfert, 1998; Brinkley, 2001). Supernumerary centrosomes tend to cluster at mitosis forming pseudo-bipolar spindles to avoid multipolar mitoses that would result in the generation of unviable progeny (Ganem et al., 2009). Formation of pseudo-bipolar spindles where merotelic chromosome attachments is frequent, is among the major causes of aneuploidy (Ganem et al., 2009). The anti-fungal drug griseofulvin was shown to freeze the process of centrosome clustering (Raab et al., 2012) and since then a number of small molecule inhibitors of this process have been synthesized and examined (Kawamura et al., 2013; Ogden et al., 2014; Bhakta-Guha et al., 2015). Centrosome declustering drugs are, however, still in pre-clinical studies (Krämer et al., 2011; Pannu et al., 2014) given two main considerations: The first is that eliminating the subpopulation of cancer cells carrying centrosome amplifications in the heterogeneous collection of cells making up a tumor is yet to be proven beneficial in anticancer therapy. The second is that identification of individuals suitable to treatment with centrosome declustering drugs still awaits routine screening methods to define the genetic makeup of patients with centrosome amplification who would benefit of such treatment (Godinho and Pellman, 2014).

#### Mitotic phosphatase inhibitors

Members of the CDC25 family of protein phosphatases act as positive regulators of CDKs that are their unique targets (see above). The only report on CDC25 targeting drugs is for phase II clinical trials initiated in 2010 with IRC 083864 under the name Debio-0931 (Lavecchia et al., 2010), a drug that has previously shown activity against pancreatic and cervical cancer xenografts (Brezak et al., 2009). To date, LB-100 is the only know drug targeting the Ser/Thr phosphatase PP2A to have entered phase I trials in combination with cytotoxic drugs or irradiation for the indication “solid tumors” (NCT01837667) (Hong et al., 2015). Inhibition of enzymes with multiple functions such as PP2A, by many considered unfeasible due to the associated high toxicity of such treatments, was shown to be well-tolerated if the drug is administered intermittently (http://www.lixte.com/Product_Development.php). LB-100 has been granted licensing in Asia for treatment of Hepatocellular Carcinoma in December 2015 (http://adisinsight.springer.com/drugs/800037966).

#### Ubiquitin-proteasome inhibitors

The established role of ubiquitin-dependent pathways in the degradation of mitotic apparatus components has made them an ideal site of intervention in cancer therapy and possible applications of proteasome inhibitors to the treatment of cancer, their mode of action and mechanisms of resistance have been amply reviewed (Crawford et al., 2011; Zhang et al., 2013). Approval of Bortezomid over a decade ago for the indications multiple myeloma and multiple cell lymphoma paved the way to the discovery of candidates with reduced side effects and improved efficacy that are currently in clinical trial (Zhang et al., 2013). Specifically to mitosis, a new perspective was provided in a report describing the use of spindle poisons in conjunction with inhibition of the ATPase activity of components of the proteasome to increase apoptosis in cancer cells (Yamada and Gorbsky, 2006), offering further possibilities of intervention.

The majority of drugs that we discussed above halt cells before mitosis or in early mitosis. Prolonged treatment with drugs interfering with microtubules dynamics has been described to lead to mitotic exit—operationally defined mitotic slippage—(Brito and Rieder, 2006), a condition that leads to the acquisition of further aneuploidy and aggressiveness (Kuukasjarvi et al., 1997; McClelland et al., 2009). Hence, significant effort has been devoted in recent years to block mitotic exit. Inhibiting the interaction of CDC20 with APC/C by TAME (tosyl-L-arginine methyl ester) has shown to effectively halt cells in mitosis and channel them to death (Zeng et al., 2010; Zeng and King, 2012). The more recently developed APC/C inhibitor Apcin, showing the ability to bind CDC20 and to prevent ubiquitylation of D-box containing APC/C targets, has provided an additional means to block mitotic exit (Sackton et al., 2014). The combined use of Apcin and TAME was reported to synergistically halt mitotic exit, hence opening new therapeutic perspectives (Sackton et al., 2014).

In a similar fashion Nutlins were described to impair physical interaction between p53 and the E3 ubiquitin ligase MDM2, promoting p53 stabilization and enhancing its tumor suppressor activity (Vassilev et al., 2004; Vassilev, 2007). Enthusiasm for these drugs, however, was mitigated by two major drawbacks: first the observation that MDM2 interacts preferentially with wild-type p53 (Lukashchuk and Vousden, 2007) and, second, the report that Nutlins exert a cytostatic effects in p53-deficient cells, indicating that they do not solely inhibit the p53/MDM2 interaction (VanderBorght et al., 2006).

In conclusion, it is foreseeable that the development of novel and specific drugs targeting components of pathways that control mitosis and/or interfere with signals that fine-tune their function, in conjunction with stratification of patients based on their genetic background, will allow to better determine combination therapies for each individual patient, taking us a step closer to personalized medicine.

## Author contributions

SF conceived the review topic and wrote the manuscript. CG contributed to write the manuscript.

## Funding

This work was supported by grants of the Promedica-Stiftung, the Stiftung zur Krebsbekämpfung and the Stiftung für wissenschaftliche Forschung.

### Conflict of interest statement

The authors declare that the research was conducted in the absence of any commercial or financial relationships that could be construed as a potential conflict of interest.
